# Multiple Sclerosis in Saudi Arabia: Clinical, Social, and Psychological Aspects of the Disease

**DOI:** 10.7759/cureus.16484

**Published:** 2021-07-19

**Authors:** Hussein Algahtani, Abdullah K Almarri, Jumanah H Alharbi, Motaz R Aljahdali, Rawan A Haimed, Rahaf Hariri

**Affiliations:** 1 Department of Medicine, Neurology Section, King Abdulaziz Medical City/King Saud Bin Abdulaziz University for Health Sciences, Jeddah, SAU; 2 Internal Medicine, Ibn Sina National College, Jeddah, SAU; 3 Neurology, Ibn Sina National College, Jeddah, SAU; 4 Medicine, Ibn Sina National College, Jeddah, SAU

**Keywords:** multiple sclerosis, depression, social, psychological, saudi arabia

## Abstract

Introduction

Multiple sclerosis (MS) is a chronic autoimmune disorder that affects the central nervous system characterized by demyelinating lesions that are disseminated in space and time. Depression is a common symptom in MS, and the pathogenesis is multifactorial. In Saudi Arabia, there is limited current literature about the incidence and relationship between depression and MS. This study is an attempt meant to address the point prevalence of depression, risk factors, and relationship to disease-modifying therapy. In addition, we describe several clinical, nutritional, and social aspects of MS.

Methods

A descriptive cross-sectional study was conducted in Jeddah, Saudi Arabia. The target sample in this study consisted of patients in Saudi Arabia with MS. Data collected included a depression questionnaire based on PHQ9, compliance on therapy, the preferred therapy, and the impact of the disease on daily activity and economic state.

Results

A total of 341 Saudi MS patients were enrolled in the present study. The gender distribution showed that 65.4% (n=223) of the study population were females. The mean age of the patients was 34.80±9.907 years. Most of the patients who were included in this study (95.6%) had depressive symptoms in variant levels. Variable changes in depression levels were detected in both genders specifically moderate depression was most common in males (33%) while females had moderately severe depression (38%). Numbers of relapses, future vision, and changes in workout were associated with statistically significant depression levels.

Conclusion

Depressive symptoms are common in patients with MS. Although all disease-modifying therapies are available in Saudi Arabia, MS clinics with multidisciplinary care are not yet efficiently activated. Non-pharmacological interventions such as smoking cessation, exercise, and psychological health should be part of the management of any patient with MS.

## Introduction

Multiple sclerosis (MS) is a chronic autoimmune disorder that affects the central nervous system (CNS), which is characterized by demyelinating lesions that are disseminated in space and time [1]. It affects young patients in the third and fourth decades of life [2], and MS in the United States showed an obvious prevalence in young women (between ages 20 and 40 years) and is one of the leading causes of disability in young adults [3]. Also, an Italian paper has talked about that the incidence in women was double that in men [4]. There are several forms of the disease. However, the most common types are relapsing-remitting (85%) and primary progressive (15%). The damage involves the brain, spinal cord, and optic nerves alone or in combination [2]. And the patient will present diverse symptoms, including sensory and motor symptoms. It also can affect bowel and bladder function as well [5]. Additionally, the patient can present with physical and psychological symptoms, especially muscle weakness, balance problems, fatigue, and other depressive symptoms [3,6]. Depressive symptoms are common in patients with MS, and the pathogenesis is multifactorial. It has influences on the general health and quality of life in patients with MS with a significant increase in the risk of suicide [7]. It generally starts after the diagnosis of MS and it is often associated with disabilities [8]. Some studies conflict that high scores of disability are related to the severity of depression [8-9], as Marrie et al. reported that MS patients with high disabilities were having a six times risk for developing depression as compared to low disability [10]. Moreover, another study demonstrated that smoking, exercise, social support, omega 3, and vitamin D supplementation were the preferred medication, and widely differentiated modifiable lifestyle factors may reduce the depression risk as a preventative [11]. Patients with MS are receiving disease-modifying therapies (DMTs) that slow the disease progression and delay the disabilities [12-13]. Its major goals are to reduce the frequency and severity of relapses or attacks, reduce lesions of the central nervous system, slow the accumulation of disability, and manage secondary symptoms [12,14]. The efficacy of DMT in MS depends on high levels of compliance. Data from managed care databases showed that the incidence of relapses increased in patients who were non-adherent or discontinued treatment with DMT [15]. Non-adherent to the treatment of MS may suggest being a major factor for the incidence of cognitive impairment and depression [16]. Depression is the main manifestation of the disease, which may be caused either by MS itself or its therapy as DMT [3,6]. On the contrary, a study reported that depressive symptoms occurrence are not likely to be associated with DMT but also some of them may decrease the incidence of depression [17]. Several studies attempted to address the correlation between MS and depression [10]. In Saudi Arabia, there is limited current literature about the incidence and relationship between depression and MS [18-19]. In a meta-analysis conducted by Etemadifar et al., there is limited availability of data with varied quality scores, diagnostic criteria, sample size, and methods [20]. Only two studies were conducted in Saudi Arabia with a small sample size (89 and 150). Those two studies were mainly discussing epidemiology and the pattern of presentation of MS in Saudi Arabia. This study is an attempt meant to address the point prevalence of depression, risk factors, and relationship to disease-modifying therapy. In addition, we describe several clinical, nutritional, and social aspects of the disease in Saudi Arabia. Furthermore, MS is common in areas far away from the equator. We would like to compare our results with the already available literature regarding the geographic distribution of the disease and report the difference if any.

## Materials and methods

A descriptive cross-sectional study was conducted in Jeddah, Saudi Arabia, starting from June to September 2020. The target sample in this study was Saudi MS patients. Patients with MS were chosen randomly through a consecutive sampling technique, which was based on eligibility criteria. Patient demographics and needed variables were collected from the patients' electronic files. Subjects were contacted by phone, and verbal consent was obtained. Data collected included the severity of depression using the Patient Health Questionnaire-9 (PHQ-9) We used the standardized, valid, and reliable Arabic version of the instrument for depression screening and diagnosis consisting of nine statements based on DSM-5. The statements are set as four-point Likert scale items and graded from 0 to 3 (not at all, several days, more than half the days, and nearly every day) to determine behavior in the past two weeks [21-22]. Each application was organized and given a score between 1 to 27 as follows: 0-4 no or minimal depression, 5-9 mild depression, 10-14 moderate depression, 15-19 moderately severe depression, and ≥20 is severe depression. An additional conclusive statement is included at the end of the diagnostic tool as a severity measure asserting, “How difficult have these problems made it for you to do your work, take care of things at home, or get along with other people?” The expected answers were not difficult at all, somewhat difficult, very difficult, and extremely difficult. Also, compliance on therapy based on the percentage of missing the doses, patients who received intravenous medications were asked if they skipped or missed any doses within the past two years, and intramuscular medications in the past six months. And the oral medications if they were missing any dose within the past three months, the preferred therapy, and the impact of the disease on daily activity and economic state. Data were entered using Microsoft Office Excel 2020 (Microsoft Corporation, Redmond, WA) and statistical analysis was performed using Statistical Package for the Social Sciences (SPSS) version 25 (IBM Corp., Armonk, NY). A p-value of <0.05 was considered statistically significant. Descriptive tests were used for quantitative data while frequency was used for qualitative data. A chi-square test was used to find the relations between the qualitative variables and to determine p-values of the relation between the presence of depression and socio-demographic variables. This study was approved by the Institutional Review Board (IRB) of King Abdullah International Medical Research Center (KAIMRC).

## Results

Demographic profile

A total of 341 Saudi MS patients were enrolled in the present study. The gender distribution showed that 65.4% (n=223) of the study population were females while 34.6% (n=118) were males, with a female to male ratio of 1.9:1. The mean age of the patients was 34.80± 9.907 years (range 15-72 years) with a mean age of the female being 34.30±9.925 years and mean age of the male being 35.75±9.84 years. Approximately, half of the participants (48.9%) had a monthly income of less than five thousand Saudi Riyal ($1,333). Other sociodemographic characteristics are detailed in Table 1.

**Table 1:** Sociodemographic characteristics

Sociodemographic characteristics	n (%)
Gender	
Male	118 (34.6%)
Female	223 (65.4%)
Age	
Years±SD; Median	34.80±9.907;33.0
Marital Status	
Single	130 (38.1%)
Married	183 (53.7%)
Divorced	25 (7.3%)
Widowed	3 (0.9%)
Income	
Less than 1000 SR	71 (20.8%)
1000-5000 SR	96 (28.1%)
5000-10000 SR	111 (32.6%)
10000-20000 SR	17 (5.1%)
20000 and More SR	46 (13.4%)
Smoking	
Smoker	91 (26.6%)
Non-smoker	250 (73.4%)
Number of Relapses	
Once in Two Years	95 (27.9%)
Once a Year	72 (21.1%)
2-4 per Year	63 (18.4%)
More Than 4 per Year	24 (7%)
No Attacks	87 (25.6%)
Future Vision	
Bad	10 (2.9%)
Not Bad	29 (8.5%)
Good	51 (15%)
Optimistic of Finding the Cure	159 (46.6%)
I Don’t Know, I Don’t Want to Predict My Future	92 (27%)
Workout	
No Change	91 (27.1%)
Fatigue With Minimal Exercise	116 (33.8%)
Missing Most of the Days With the Same Exercise	20 (5.8%)
Same Number of Days With Minimizing Usual Exercise	23 (6.7%)
Unable To Do Any Exercise	68 (19.8%)
I Don’t Exercise	23 (6.7%)
Percentage of Compliance	
Less Than 30% of the Doses	42 (12.3%)
30% to 60% of the Doses	21 (6.2%)
More than 60% of the Dose	17 (5%)
I Don’t Miss Any Dose	261 (76.5%)

Clinical data of the participants

**Figure 1:**
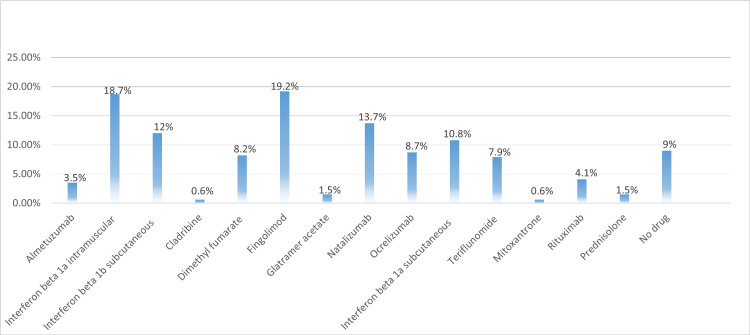
Drugs used by participants

**Figure 2:**
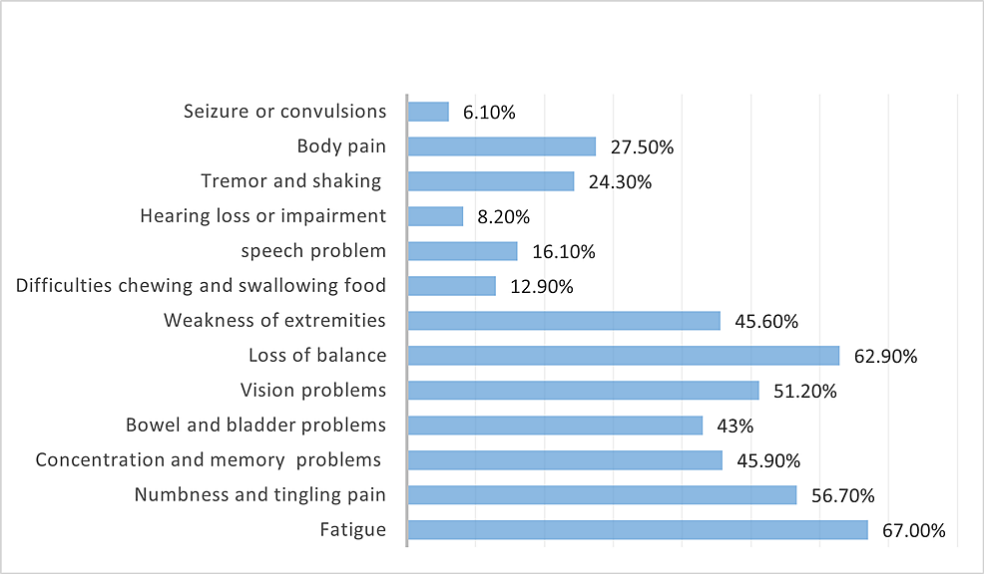
Most common symptoms

Regarding the number of relapses in the last four years, 25.6% (n=87) of the patients did not experience any relapses. Most of the patients (91%) use DMT. However, fingolimod and interferon beta 1a intramuscular injections were reported as the highest drugs used by participants with 19.4% (n=66) and 18.7% (n=63), respectively. A total of 261 patients (76.5%) were committed to dosages and did not miss any. Fatigue was the most common symptom present in the MS patients 67% (n=229). Only 6.7% (n=23) did not exercise at all. An optimistic view was reported by 46.6% (n=159) of the participants for finding the cure for MS. Additional information is specified in Table 1 and Figures 1-2.

Depression symptoms

Most of the patients who are included in this study (95.6%) had depressive symptoms in variant levels as shown in Table 2, where the most frequent level was moderately severe depression (34.1%). Table 3 is showing other levels. The presence of depressive symptoms was not statistically significant with gender (p-value = 0.552) and age group (p-value = 0.735). While the numbers of relapses in the last four years were statistically significantly associated with the presence of depressive symptoms (p-value = 0.011). In addition, changes in workouts were statistically significantly associated with the presence of depressive symptoms (p-value = 0.006). Furthermore, fatigue, as well as bowel and bladder symptoms, were statistically significantly associated with the presence of depressive symptoms (p-value = 0.012) (p-value = 0.004). Ninety-two point one percent (92.1%) of the participants who were using interferon beta 1a intramuscular injections had depressive symptoms, but no statistically significant difference was detected. Additional information is specified inTable 4*.*

**Table 2:** Presence of depressive symptoms

Presence of depressive symptoms	n (%)
None or Minimal	13 (3.8%)
Yes	328 (95.6%)

**Table 3:** Depression levels

Depression levels	n (%)
Minimal depression (1-4)	13 (3.8%)
Mild depression (5-9)	55 (16%)
Moderate depression (10-14)	115 (33.5%)
Moderately severe depression (15-19)	117 (34.1%)
Severe depression (20-27)	41 (12%)

**Table 4:** Additional information on the depression occurrence in our study population

Additional information on depression occurrence in our study population				
Demographic Variable	Distribution	Depression		P-Value
		No	Yes	
Gender	Male	6 (1.8%)	112 (32.8%)	0.552
	Female	7 (2.1%)	216 (63.3%)	
Age group	20 and less	1 (0.3%)	14 (4.1%)	0.735
	21-30	3 (0.9%)	114 (33.4%)	
	31-40	6 (1.8%)	119 (34.9%)	
	41-50	2 (0.6%)	54 (15.8%)	
	50 and more	1 (0.3%)	27 (7.9%)	
Marital status	Single	5 (1.5%)	127 (37.2%)	0.919
	Married	8 (2.3%)	173 (50.7%)	
	Widow	0 (0.0%)	3 (0.9%)	
	Divorced	0 (0.0%)	25 (7.3%)	
Smoker	Smoker	7 (2.1%)	243 (71.3%)	0.113
	Non-smoker	6 (1.7%)	85 (24.9%)	
How many relapses	Once in two years	3 (0.9%)	92 (27.0%)	0.011
	Once a year	1 (0.3%)	71 (20.8%)	
	2-4 per year	0 (0.0%)	63 (18.5%)	
	More than 4 per year	0 (0.0%)	24 (7.0%)	
	No relapses	9 (2.6%)	78 (22.9%)	
Future vision	Bad	0 (0.0%)	10 (2.9%)	0.025
	Not bad	0 (0.0%)	29 (8.5%)	
	Good	5 (1.5%)	46 (13.5%)	
	Optimistic of finding the cure	8 (2.3%)	151 (44.3%)	
	I don’t know, I don’t want to predict my future	0 (0.0%)	92 (27.0%)	
Workout	No change	10 (2.9%)	81 (23.8%)	0.006
	Laziness with minimized exercise	2 (0.6%)	114 (33.4%)	
	Missing most of the days with the same exercise	0 (0.0%)	20 (5.9%)	
	Same number of days with minimizing usual exercise	0 (0.0%)	23 (6.7%)	
	Unable to do any exercise	0 (0.0%)	68 (19.9%)	
	I don’t exercise	1 (0.3%)	22 (6.5%)	
Percentage of compliance	Less than 30% of the doses	2 (0.6%)	40 (11.7%)	0.621
	30 to 60% of the doses	0 (0%)	21 (6.1%)	
	More than 60%of the dose	1 (0.3%)	16 (4.9%)	
	I don’t miss any dose	10 (2.8%)	251 (73.6%)	
Drugs	Alemtuzumab	0 (0.0%)	12 (3.5%)	1
	Interferon beta 1a intramuscular	5 (1.5%)	58 (17.0%)	0.071
	Interferon beta 1b subcutaneous	2 (0.6%)	39 (11.4%)	0.661
	Cladribine	0 (0.0%)	2 (0.6%)	1
	Dimethyl fumarate	0 (0.0%)	28 (8.2%)	0.611
	Fingolimod	3 (0.9%)	63 (18.5%)	0.722
	Glatiramer acetate	0 (0.0%)	5 (1.5%)	1
	Natalizumab	3 (0.9%)	44 (12.9%)	0.4
	Ocrelizumab	0 (0.0%)	30 (8.8%)	0.615
	Interferon beta 1a subcutaneous	0 (0.0%)	36 (10.6%)	0.376
	Teriflunomide	0 (0.0%)	27 (7.9%)	0.61
	Mitoxantrone	0 (0.0%)	2 (0.6%)	1
	Rituximab	0 (0.0%)	14 (4.1%)	1
	No drug	0 (0.0%)	31 (9.1%)	0.617
	Prednisolone	0 (0.0%)	5 (1.5%)	1
Symptoms	Fatigue	4 (1.2%)	225 (66%)	0.012
	Numbness and tingling pain	3 (0.9%)	190 (55.7%)	0.028
	Concentration and memory problems	2 (0.6%)	154 (45.2%)	0.05
	Bowel and bladder problems	0 (0.0%)	147 (43.1%)	0.004
	Vision problems	6 (1.8%)	168 (49.3%)	0.94
	Loss of balance	5 (1.5%)	210 (61.6%)	0.079
	Weakness of extremities	3 (0.9%)	153 (44.9%)	0.165
	Difficulties chewing and swallowing food	0 (0%)	44 (12.9%)	0.388
	Speech problem	0 (0%)	55 (16.1%)	0.139
	Hearing loss or impairment	0 (0%)	28 (8.2%)	0.611
	Tremor and shaking	1 (0.3%)	82 (24%)	0.201
	Body pain	1 (0.3%)	93 (27.3%)	0.123
	Seizure or convulsions	0 (0%)	21 (6.2%)	1

Depression levels

Variable changes in depression levels were detected in both genders (p-value = 0.92). However, moderate depression was most common in males (33%) while females had moderately severe depression (38%). Moderately severe depression was the highest in the second age group 21-30 (39.6%) (p-value = 0.351). Numbers of relapses, future vision, and changes in workout were associated with statistically significant depression levels (p-value <0.001). There was no relationship between drug compliance (missing doses) and depression. Teriflunomide and cladribine use were associated with statistically significant depression levels (p-value = 0.022 and p-value = 0.037, respectively). The neurological symptoms associated with a statistically significant difference were fatigue, concentration and memory problems, bowel and bladder symptoms, weakness of extremities, tremor and shaking, and speech problems (p-value <0.001). Detailed information is specified in Table 5*.*

**Table 5:** Detailed information on the depression levels in our study population DMT: disease-modifying therapy

Detailed information on the depression levels in our study population							
Demographic Variable	Distribution		Depression Levels				P-Value
		Minimal	Mild	Moderate	Moderately severe	Severe	
Gender	Male	6 (1.8%)	24 (7.0%)	39 (11.4%)	31 (9.1%)	18 (5.3%)	0.92
	Female	7 (2.1%)	31 (9.1%)	76 (22.3%)	86 (25.2%)	23 (6.7%)	
Age group	20 and less	1 (0.3%)	4 (1.2%)	4 (1.2%)	3 (0.9%)	3 (0.9%)	0.351
	21-30	3 (0.9%)	12 (3.5%)	40 (11.7%)	46 (13.5%)	16 (4.7%)	
	31-40	6 (1.8%)	22 (6.5%)	39 (11.4%)	42 (12.3%)	16 (4.7%)	
	41-50	2 (0.6%)	11 (3.2%)	24 (7.0%)	17 (5.0%)	2 (0.6%)	
	50 and more	1 (0.3%)	6 (1.8%)	8 (2.3%)	9 (2.6%)	4 (8.2%)	
Marital status	Single	5 (1.5%)	18 (5.3%)	43 (12.6%)	46 (13.5%)	20 (5.9%)	0.862
	Married	8 (2.3%)	30 (8.8%)	64 (18.8%)	60 (17.6%)	19 (5.6%)	
	Widow	0 (0.0%)	7 (2.1%)	7 (2.1%)	9 (2.6%)	2 (0.6%)	
	Divorced	0 (0.0%)	0 (0.0%)	1 (0.3%)	2 (0.6%)	0 (0.0%)	
Smoker	Smoker	6 (1.8%)	10 (3.0%)	31 (9.0%)	35 (10.3%)	9 (2.7%)	0.275
	Non smoker	7 (2.0%)	45 (13.1%)	84 (24.7%)	82 (24%)	32 (9.4%)	
How many relapses	Once in two years	3 (0.9%)	15 (4.4%)	39 (11.4%)	28 (8.2%)	10 (2.9%)	<0.001
	Once a year	1 (0.3%)	13 (3.8%)	23 (6.7%)	27 (7.9%)	8 (2.3%)	
	2 4 per year	0 (0.0%)	6 (1.8%)	15 (4.4%)	29 (8.5%)	13 (3.8%)	
	More than 4 per year	0 (0.0%)	3 (0.9%)	3 (0.9%)	13 (3.8%)	5 (1.5%)	
	No relapses	9 (2.6%)	18 (5.3%)	35 (10.3%)	20 (5.9%)	5 (1.5%)	
Future vision	Bad	0 (0.0%)	1 (0.3%)	1 (0.3%)	5 (1.5%)	3 (0.9%)	<0.001
	Not bad	0 (0.0%)	2 (0.6%)	9 (2.6%)	14 (4.1%)	4 (1.2%)	
	Good	5 (1.5%)	13 (3.8%)	17 (5.0%)	11 (3.2%)	5 (1.5%)	
	Optimistic of finding the cure	8 (2.3%)	32 (9.4%)	59 (17.3)	48 (14.1%)	12 (3.5%)	
	I don’t know, I don’t want to predict my future	0 (0.0%)	7 (2.1%)	29 (8.5%)	39 (11.4%)	17 (5.0%)	
Workout	No change	10 (2.9%)	24 (7.0%)	37 (10.9%)	17 (5.0%)	3 (0.9%)	<0.001
	Laziness with minimized exercise	2 (0.6%)	17 (5.0%)	44 (12.9%)	45 (13.2%)	8 (2.3%)	
	Missing most of the days with the same exercise	0 (0.0%)	0 (0.0%)	9 (2.6%)	9 (2.6%)	2 (0.6%)	
	Same number of days with minimizing usual exercise	0 (0.0%)	3 (0.9%)	5 (1.5%)	10 (2.9%)	5 (1.5%)	
	Unable to do any exercise	0 (0.0%)	6 (1.8%)	13 (3.8%)	26 (7.6%)	23 (6.7%)	
	I don’t exercise	1 (0.3%)	5 (1.5%)	7 (2.1%)	10 (2.9%)	0 (0.0%)	
Percentage of compliance	Less than 30% of the doses	2 (0.6%)	10 (3.2%)	15 (4.8%)	10 (3.2%)	5 (1.6%)	0.423
	30 to 60% of the doses	0 (0.0%)	2 (0.6%)	3 (1.0%)	12 (3.8%)	4 (1.3%)	
	More than 60%of the dose	1 (0.3%)	3 (1.0%)	6 (1.9%)	6 (1.9%)	1 (0.3%)	
	I don’t miss any dose	10 (2.9%)	40 (11.7%)	91 (26.7%)	89 (26.1%)	31 (9.6%)	
Drug	Alemtuzumab	0 (0.0%)	1 (0.3%)	4 (1.2%)	5 (1.5%)	2 (0.6%)	0.926
	Interferon beta 1a intramuscular	5 (1.5%)	12 (3.5%)	23 (6.7%)	20 (5.9%)	3 (0.9%)	0.096
	Interferon beta 1b subcutaneous	2 (0.6%)	5 (1.5%)	14 (4.1%)	16 (4.7%)	4 (1.2%)	0.889
	Cladribine	0 (0.0%)	0 (0.0%)	0 (0.0%)	0 (0.0%)	2 (0.6%)	0.037
	Dimethyl fumarate	0 (0.0%)	4 (1.2%)	7 (2.1%)	11 (3.2%)	6 (1.8%)	0.425
	Fingolimod	3 (0.9%)	14 (4.1%)	28 (8.2%)	18 (5.3%)	3 (0.9%)	0.063
	Glatiramer acetate	0 (0.0%)	0 (0.0%)	1 (0.3%)	1 (0.3%)	3 (0.9%)	0.082
	Natalizumab	3 (0.9%)	4 (1.2%)	18 (5.3%)	15 (4.4%)	7 (2.1%)	0.378
	Ocrelizumab	0 (0.0%)	2 (0.6%)	10 (2.9%)	12 (3.5%)	6 (1.8%)	0.312
	Interferon beta 1a subcutaneous	0 (0.0%)	3 (0.9%)	15 (4.4%)	14 (4.1%)	4 (1.2%)	0.478
	Teriflunomide	0 (0.0%)	4 (1.2%)	3 (0.9%)	16 (4.7%)	4 (1.2%)	0.022
	Mitoxantrone	0 (0.0%)	0 (0.0%)	2 (0.6%)	0 (0.0%)	0 (0.0%)	0.651
	Rituximab	0 (0.0%)	2 (0.6%)	3 (0.9%)	6 (1.8%)	3 (0.9%)	0.643
	Not on DMT	0 (0.0%)	7 (2.1%)	11 (3.2%)	9 (2.6%)	4 (1.2%)	0.72
	Prednisolone	0 (0.0%)	0 (0.0%)	1 (0.3%)	3 (0.9%)	1 (0.3%)	0.615
Symptoms	Fatigue	4 (1.2%)	21 (6.2%)	79 (23.2%)	88 (25.8%)	37 (10.9%)	<0.001
	Numbness and tingling pain	3 (0.9%)	31 (9.1%)	67 (19.6%)	66 (19.4%)	26 (7.6%)	0.144
	Concentration and memory problems	2 (0.6%)	9 (2.6%)	48 (14.1%)	67 (19.6%)	30 (8.8%)	<0.001
	Bowel and bladder problems	0 (0.0%)	15 (4.4%)	47 (13.8%)	63 (18.5%)	22 (6.5%)	<0.001
	Vision problems	6 (1.8%)	26 (7.6%)	51 (15.0%)	67 (19.6%)	24 (7.0%)	0.246
	Loss of balance	5 (1.5%)	26 (7.6%)	68 (19.9%)	87 (25.5%)	29 (8.5%)	0.001
	Weakness of extremities	3 (0.9%)	12 (3.5%)	48 (14.1%)	65 (19.1%)	28 (8.2%)	<0.001
	Difficulties chewing and swallowing food	0 (0.0%)	1 (0.3%)	11 (3.2%)	25 (7.3%)	7 (2.1%)	0.001
	Speech problem	0 (0.0%)	2 (0.6%)	13 (3.8%)	29 (8.5%)	11 (3.2%)	<0.001
	Hearing loss or impairment	0 (0.0%)	0 (0.0%)	6 (1.8%)	15 (4.4%)	7 (2.1%)	0.003
	Tremor and shaking	1 (0.3%)	3 (0.9%)	24 (7.0%)	39 (11.4%)	16 (4.7%)	<0.001
	Body pain	1 (0.3%)	8 (2.3%)	30 (8.8%)	39 (11.4%)	16 (4.7%)	0.015
	Seizure or convulsions	0 (0.0%)	0 (0.0%)	6 (1.8%)	9 (2.6%)	6 (1.8%)	0.035

## Discussion

This study attempted to assess the clinical, social, and psychological aspects of MS. We examined a cohort of MS patients to assess their profile and determine the relationship between depression and disability worsening. We included a total of 341 patients, almost two-thirds of them being females in their fourth decade of life. Regarding the female predominance of this disease, our study confirmed this finding, which is similar to other countries across the world [23]. The sociodemographic characteristics, including monthly income, are similar to other MS publications from Saudi Arabia [7].

In our study, most of the patients were using disease-modifying therapy (91%). The two most common medications prescribed were fingolimod and interferon beta 1a intramuscular injections. Although medication adherence was not assessed, 76.5% were committed to dosages and did not miss any of their medication. This observation is different from that of Alhazzani et al. [24], who concluded that medication adherence among MS patients in Saudi Arabia is low.

Smoking has been shown to have a strong association with MS in several studies [25].In our study, one-quarter of our patients were smokers of either cigarettes or waterpipes. Although further studies are needed, it seems that the incidence of smoking among MS patients is slightly higher than similar age group people without MS (normal population). The prevalence of current smoking in the Saudi community is 11.6%. In Saudi Arabia, there are gender differences since smoking among males was 27 times those among females [26]. In our studies, smoking is only two times more common in males than females. These findings indicate that effective tobacco-control programs for both males and females with different intervention strategies must be developed and implemented for patients with MS.

In our survey, more than half of the patients had no relapses (25.6%), one relapse in two years (27.9%), or one relapse annually (20.1%). Around one-quarter of the patients had either two to four relapses per year (18.4%) or more than four relapses annually (7%).

The future vision of patients in our study was optimistic or good in more than half of the patients surveyed. Only 2.9% had a bad future vision, and 27% do not know or do not want to predict the future. Our study confirmed the finding of Alhazzani et al. [24] that medication adherence is associated with higher treatment satisfaction since most of the surveyed patients were adherent to treatment. More work should be done with multidisciplinary care, including a psychologist and social worker, to educate the patient about the disease and the presence of highly effective disease-modifying therapy with excellent control of disease activity. This will reflect in a better optimistic view on the future.

Exercise has the ability to improve the mental, emotional, and, to a lesser extent, the physical charts of symptomatology in patients with MS. In addition, exercise reduces the predisposition to cardiovascular and metabolic disorders [27]. In our study, around 6.7% of patients do not exercise, and 27.1% continue to exercise with no effect of the disease on the quality or quantity of the exercise. Unfortunately, 33.8% have fatigue with minimal exercise, and 12.5% are either missing days or minimizing exercise. Around one-fifth of the patients are unable to do any exercise. Physical exercise is an important non-pharmacological instrument in patients with MS. Education about the importance of exercise should be disseminated and solutions for the reasons behind the lack of exercise such as fatigue should be addressed.

Depressive spectrum disorders are quite high in patients diagnosed with MS. The point prevalence rates for major depressive syndromes in patients with MS visiting outpatient clinics were 14% and the lifetime risk for major depressive disorder in the MS population may be as high as 50% [28]. In our study, 95.6% of the sample surveyed had depressive symptoms. Only one-fifth of these depressive symptoms were minimal (3.8%) or mild (16%) and the rest were moderate depression (33.5%), moderately severe (34.1%), and severe (12%). Gender, age group, marital status, and smoking have no or minimal effect on the rate of depression. Future vision, whether bad, good, or optimistic, had no relation to the frequency of occurrence of depression. However, those who had a bad future vision had a severe form of depression. In addition, patients who had frequent attacks had a significantly higher percentage of depression. For that, psychological care, including psychiatric consultation, should be practiced in all neurology clinics treating patients with multiple sclerosis.

Over the past two decades, tremendous advances in the treatment of MS have occurred, and currently, there are more than 17 disease-modifying therapies available [29]. In our study, the occurrence of depression had no relationship to the type of disease-modifying therapy used. This may indicate that the occurrence of depression may have a relationship with the disease itself and other factors such as the level of physical disability but not the type of disease-modifying therapy. When depression occurs, it is more severe with two disease-modifying therapies: cladribine and teriflunomide. Further studies should be tackling this issue in Saudi Arabia.

In our study, four symptoms were noticed to be associated with more incidence of depression. These are fatigue, sensory symptoms, cognitive dysfunction, and bowel and bladder problems. More moderate to severe depression was noticed in the previous four complaints with the exception of sensory symptoms. In addition, most symptoms, once they exist, cause the depression to be more significant with the only exception being visual impairment. We could not explain this finding.

## Conclusions

Depression is a very common disorder in patients with MS. Although all disease-modifying therapies are available in Saudi Arabia, MS clinics with multidisciplinary care are not yet efficiently activated. Non-pharmacological interventions, such as smoking cessation, exercise, and psychological health, should be part of the management of any patient with MS. Aggressive management of symptoms, with a special focus on fatigue, sensory symptoms, cognitive dysfunction, and bowel and bladder problems, should be established. MS is a disease affecting the young generation in their third and fourth decades, with a huge burden on their health, which necessitates conducting further studies, especially in the presence of an extremely supportive and freely provided healthcare system.
